# Involvement of brain-derived neurotrophic factor in time-dependent neurodegeneration in the murine superior colliculus after intravitreal injection of *N*-methyl-D-aspartate

**Published:** 2009-04-03

**Authors:** Hirotaka Tanaka, Yasushi Ito, Shinsuke Nakamura, Masamitsu Shimazawa, Hideaki Hara

**Affiliations:** 1 Department of Biofunctional Evaluation, Molecular Pharmacology, Gifu Pharmaceutical University, Gifu, Japan; 2 Molecular Imaging Research Program, Riken, Kobe, Japan

## Abstract

**Purpose:**

To clarify the effects on the visual pathway that occur following retinal damage, we examined the morphological alterations present in the superior colliculus (SC) after *N*-methyl-D-aspartate (NMDA)-induced retinal damage in mice.

**Methods:**

NMDA was injected into the vitreous body of the left eye in mice to induce retinal damage. The time-dependent neuronal degeneration in the SC was assessed using immunohistochemistry.

**Results:**

The number of neuronal nuclear specific protein (NeuN)-immunostained neurons showed a significant decrease in the contralateral SC at both 90 and 180 days after intravitreal NMDA injection. In contrast, the ipsilateral SC displayed no significant change in the number of NeuN-positive cells. An increase in glial fibrillary acid protein (GFAP) immunoreactivity was observed in the contralateral SC at 7, 30, and 90 days after NMDA injection and in the ipsilateral SC at 7 days, while brain-derived neurotrophic factor (BDNF) expression was increased in the contralateral SC at 30 and 90 days. In the contralateral SC, some GFAP-positive astroglial cells also exhibited BDNF at 30 days after NMDA injection.

**Conclusions:**

Evidence of time-dependent morphological neuronal degeneration along the retinocollicular pathway from the retina to the SC was detected at 90 and 180 days, but not at 30 days, after NMDA-induced retinal damage. This neurodegeneration was preceded by an increase in BDNF expression in the SC, specifically at 30 and 90 days after NMDA injection. Hence, these findings may provide useful information concerning the pathological mechanisms of several disorders accompanied by retinal degeneration.

## Introduction

The superior colliculus (SC), a major target of primary optic projections, plays an important role in guiding the movements of the eye, ear, and head [1,2]. In the SC, there are three main types of sites: the superficial, intermediate, and deep layers. In some species, the superficial layer receives input from both the contralateral and the ipsilateral retinas [3], although in most mammalian species the projection to the superficial layer is from the contralateral retina. At least 70% of retinal ganglion cells (RGC) have been found to project to the SC in mice [4]. Since the superficial layer of the SC is innervated by axons from the retina, their input is almost exclusively related to vision.

RGC death is a common feature of many ophthalmic disorders, such as glaucoma, optic neuropathies, and various retinovascular diseases (diabetic retinopathy and retinal vein occlusions). RGC death may occur via a variety of mechanisms involving, for example, reactive oxygen species [5], excitatory amino acids [6], nitric oxide [7], and apoptosis [8].

Glutamate is the principal excitatory neurotransmitter in the central nervous system and retina. Excessive activation of glutamate receptors by glutamate released from injured RGC is implicated in the glaucomatous RGC death process [9]. These findings indicate that glutamate is associated with damage to the eye and brain. In a recent study on mice, we found that the cell number in the ganglion cell layer decreased and retinal damage occurred within seven days after intravitreal NMDA injection; at the same time, the number of neurons in the lateral geniculate nucleus, the major relay center between the eye and the visual cortex, decreased at 90 days after injection [10]. However, to our knowledge, no previous investigation has been made of time-dependent alterations along the collicular visual pathways after retinal injury in mice. Clarification of this pathogenesis could help to elucidate the mechanism responsible for blindness caused by various ophthalmic disorders (e.g., glaucoma).

Neurotrophins, originally identified for their ability to promote neuronal survival and differentiation, are potent modulators of synaptic connectivity in the central nervous system, influencing synaptic structure and function [11,12]. Specifically, BDNF influences the morphological complexity of axons and dendrites [13-15], modulates synapse maturation [16,17], increases the number of synapses in the developing brain [18,19], and controls the ultrastructural composition of synapses [20,21]. Thus, BDNF is involved in multiple aspects of synaptogenesis, from formation to the functional maturation of synapses. Reportedly, endogenous BDNF in the dorsal root ganglia and spinal cord is required for the enhanced regeneration of ascending sensory neurons after conditioning lesions of the sciatic nerve, and peripherally applied BDNF may have therapeutic effects on spinal cord injuries [22]. In our previous study, intravitreal injection of BDNF inhibited RGC damage after axotomy [23]. However, to our knowledge, there are no reports regarding the role of BDNF in the subsequent neuron loss in the SC after retinal damage. We therefore designed the present study to examine the time-dependent morphological and immunohistochemical alterations and changes in BDNF expression occurring in the murine SC after intravitreal injection of NMDA.

## Methods

### Animals

Male adult (6 week-old) C57BL/6J mice weighing 20−32 g (Japan Clea, Inc. Fujimiya, Japan) were kept under lighting conditions of 12 h light and 12 h dark. The house or the cage was 17×30×14 cm, food from CE-2 (Japan Clea, Inc. Fujimiya, Japan), and 500 ml/8 mice/week amount of water was given to mice.

All experiments were performed in accordance with the ARVO Statement for the Use of Animals in Ophthalmic and Vision Research and were approved and monitored by the Institutional Animal Care and Use Committee of Gifu Pharmaceutical University.

### NMDA Injection

**Figure 1:**
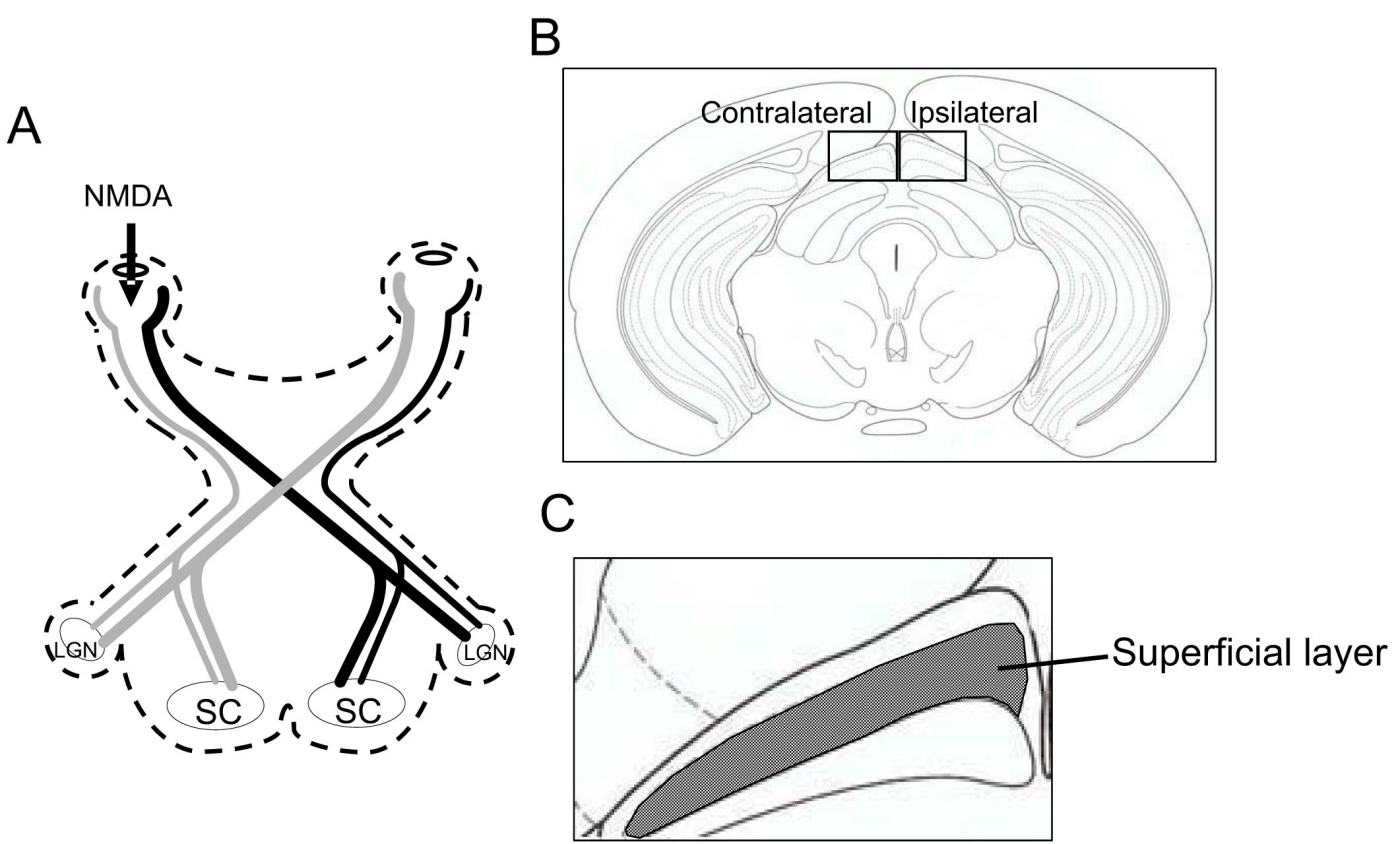
The projection pathway from the retina to the SC. Illustrations show (**A**) the pathway from the retina to the superior colliculus (SC), (**B**) the coronal section through the level of the SC (bregma−3.40 mm) in mice; boxed areas contain the SC of the contralateral side and the ipsilateral side and (**C**) the superficial layer of the SC.

Mice were anesthetized (46 mice) with 3.0% isoflurane (Merck, Osaka, Japan) and maintained with 1.5% isoflurane in 70% N_2_O and 30% O_2_ via an animal general anesthesia apparatus (Soft Lander; Sin-ei Industry Co. Ltd., Saitama, Japan). Retinal damage was induced by intravitreal injection of 2 µl NMDA (Sigma-Aldrich, St. Louis, MO) dissolved at 20 mM in 0.01 M phosphate-buffered saline (PBS; containing 2.7 mM KCl, 1.5 mM KH_2_PO_4_, 136.8 mM NaCl, and 8.1 mM Na_2_HPO_4_-12H_2_O) at pH 7.4. This was injected into the vitreous body of the left eye while the mouse was under anesthesia (Figure 1). One drop of levofloxacin ophthalmic solution (Santen Pharmaceuticals Co. Ltd., Osaka, Japan) was applied topically to the treated eye immediately after the intravitreal injection. NMDA-treated mice were euthanized at 3 (n=5), 7 (n=4), 30 (n=6), 90 (n=5), and 180 days (n=11). The control (untreated) group (n=13) consisted of mice euthanized at 1 (n=4), 30 (n=3), 90 (n=3), and 180 days (n=3). The sham group was composed of mice who received intravitreal injection of PBS instead of NMDA. This group was euthanized at 3 (n=3), 7 (n=3), 30 (n=3), 90 (n=3), and 180 days (n=3).

### Sample preparation

At the end of the assigned survival periods, mice were euthanized with an intraperitoneal injection of 80 mg/kg sodium pentobarbital (Nembutal; Dainippon, Osaka, Japan) and perfused with 4% (w/v) paraformaldehyde solution in 0.01 M PBS (except for the immunohistochemistry of BDNF, when 2% [w/v] paraformaldehyde solution in 0.01 M PBS was used). The brains were removed after a 15 min perfusion at 4 °C, immersed in the same fixative solution for 24 h, soaked in 25% (w/v) sucrose for 1 day, and then frozen in embedding compound (Tissue-Tek; Sakura Finetechnical Co. Ltd., Tokyo, Japan). Coronal sections through the SC (bregma −3.40 mm) were cut in 10 µm thicknesses (Figure 1).

### Immunohistochemistry

For immunohistochemistry, coronal sections containing the SC were placed on slides (Mascoat; Matsunami, Osaka, Japan), washed with 0.01 M PBS, and then treated with 0.3% hydrogen peroxidase in methanol for 30 min at room temperature. Next, the sections were blocked with mouse-on-mouse blocking reagent (M.O.M. immunodetection kit; Vector Laboratories, Inc., Burlingame, CA) and then incubated either with 1: 250 dilution mouse anti-NeuN monoclonal antibody (Chemicon, Temecula, CA) or with mouse anti-GFAP monoclonal antibody (Ylem, Rome, Italy) for 1 day at 4 °C. They were washed with 0.01 M PBS and then incubated with biotinylated anti-mouse IgG before being incubated with the avidin-biotin-peroxidase complex for 30 min at room temperature, and finally visualized using diamino benzidine (DAB) as a peroxidase substrate (Vector Laboratories).

In the immunostaining procedures for BDNF, coronal sections containing the SC were washed with 0.01 M PBS containing 0.05% Tween-20 and then treated with 0.3% hydrogen peroxidase in methanol. Next, they were preincubated with 10% normal goat serum (Vector) in 0.01 M PBS for 30 min and then incubated for one day at 4 °C with 1:1,000 specific rabbit anti-BDNF polyclonal antibody [24] in the following solution: 10% normal goat serum in 0.01 M PBS containing 0.3% (v/v) Triton X-100. They were washed with 0.01 M PBS containing 0.05% Tween-20 and then incubated with biotinylated anti-rabbit IgG before being incubated with the avidin-biotin-peroxidase complex for 30 min at room temperature, and finally visualized using DAB as a peroxidase substrate.

To visualize the colocalization of BDNF with NeuN or GFAP, we performed double immunofluorescence on the SC sections from the NMDA-treated mice. Coronal sections containing the SC were washed with 0.01 M PBS and 0.05% Tween-20 then treated with 0.3% hydrogen peroxidase in methanol for 30 min at room temperature. Next, they were preincubated with 10% normal goat serum in PBS for 30 min and then incubated overnight at 4 °C with 1:1,000 dilution rabbit anti-BDNF monoclonal antibody in the following solution: 10% normal goat serum in PBS with 0.3% (v/v) Triton X-100. The sections were blocked with M.O.M. blocking reagent and incubated either with 1:250 mouse anti-NeuN monoclonal antibody (Chemicon) or with 1:250 mouse anti-GFAP monoclonal antibody (Ylem) for 1 day at 4 °C. They were washed with 0.01 M PBS containing 0.05% Tween-20 and incubated for 3 h at room temperature with a mixture of 1:1,000 dilution Alexa Fluor 488 F(ab’)_2_ fragment of goat anti-rabbit IgG (H^+^L; Molecular Probes, Eugene, OR) and 1:1,000 dilution Alexa Fluor 488 F(ab’)_2_ fragment of goat anti-mouse IgG (H^+^L; Molecular Probes).

Total images of the SC were taken using a microscope (BX50; Olympus, Tokyo, Japan) fitted with 10X and 40X microscope objective lenses. The images visualized by DAB were taken using a digital camera (Coolpix 4500; Nikon, Toyko, Japan), and immunofluorescence images were taken using a charge-coupled device camera (DP30BP; Olympus) at 1,360×1,024 pixels via Metamorph (Universal Imaging Corp., Downingtown, PA). NeuN and BDNF-positive cells and GFAP-positive astroglial cells were counted in the superficial layer of the SC by a single observer (H.T.), as in our previous report [10]. Counted sections were shown in Figure 1 (bregma −3.40 mm and 10 µm thickness). Data were counted and averaged using three sections of each animal. The results were expressed as positive cells per 1 mm^2^.

### Data analysis

Data are presented as means±SEM. Statistical comparisons (one-way ANOVA followed by a Dunnett’s test) were made using STAT View version 5.0 (SAS Institute, Inc., Cary, NC). A value of p<0.05 was considered to indicate statistical significance.

## Results

### Neuronal Cells Immunostained for NeuN

**Figure 2:**
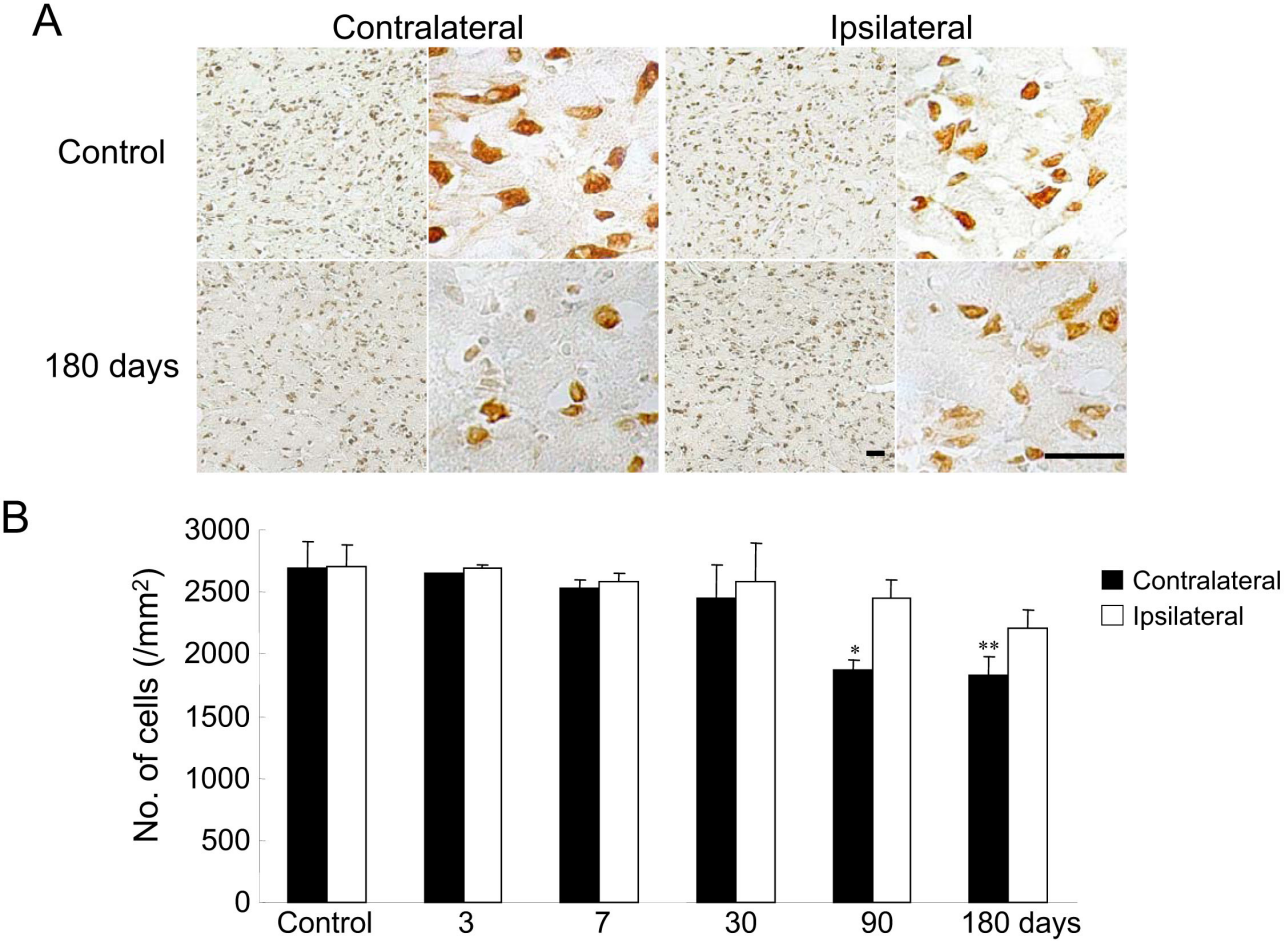
NeuN immunostaining of sections of the SC. **A:** Representative microphotographs of the superior colliculus (SC) are shown for the control group (untreated) for 180 days after *N*-methyl-D-aspartate (NMDA) injection (the contralateral side and the ipsilateral side). The scale bars represent 30 µm. **B:** The average number of neuronal nuclear specific protein (NeuN)-labeled neurons was counted in the SC. Each value represents the mean±SEM (n=4–11). The asterisk indicates p<0.05, while the double asterisk represents p<0.01 versus control (untreated mice; Dunnett’s test).

NeuN, an antibody known as a neuron-specific antigen, selectively and clearly stains neuronal perikarya and nuclei [25]. NeuN and Nissl staining produce highly correlated estimates of neuronal density, size, and shape [26]. Furthermore, NeuN may be particularly useful when it is important to distinguish small neurons from glial cells. In our control mice, NeuN-positivity was seen in almost all neurons within the superficial layer of the SC (Figure 2A). In the NMDA-treated group, NeuN-labeled neurons were first seen to be significantly decreased in number in the contralateral SC at 90 days after NMDA injection; the number decreased to 69.2% (90 days) and 68.1% (180 days) of control after NMDA injection (Figure 2B). In contrast, no significant decrease in the number of NeuN-labeled neurons (versus control) was observed in the superficial layer of the ipsilateral SC at 3, 7, 30, 90, and 180 days (Figure 2B). Furthermore, no morphological differences in the SC were observed among the control and sham-treated mice (data not shown).

### Astrocytes Immunostained for GFAP

**Figure 3:**
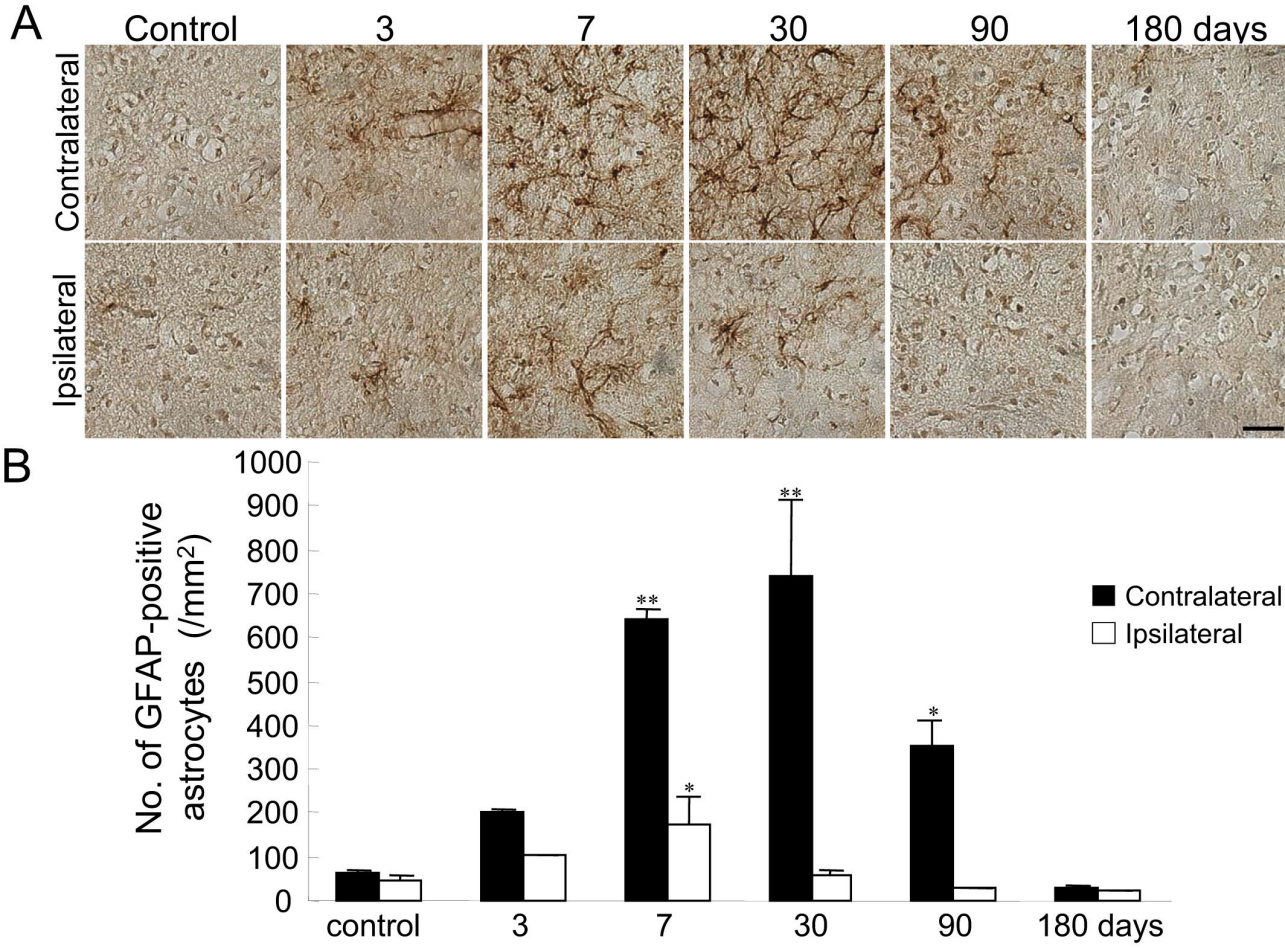
GFAP-positive astrocyte of sections of the SC. **A:** Representative microphotographs of the superior colliculus (SC; the contralateral side and the ipsilateral side) are shown for the control group (untreated) 3, 7, 30, 90, and 180 days after *N*-methyl-D-aspartate (NMDA) injection. The scale bar represents 30 µm. **B:** The average number of glial fibrillary acid protein (GFAP)-positive astrocytes was counted in the SC. Each value represents the mean±SEM (n=3). The asterisk indicates p<0.05, while the double asterisk represents p<0.01 versus control (untreated mice; Dunnett’s test).

Activation of astrocytes could be easily detected by staining with antibodies against GFAP. We investigated the time-dependent alterations of activated astrocytes in the superficial layer of the SC by immunohistochemical analysis (Figure 3) At 3, 7, 30, and 90 days and 3, 7, and 30 days after NMDA injection, GFAP expression was observed in the contralateral and ipsilateral SC, respectively (Figure 3A). Quantitative analysis of GFAP-positive astroglial cells is shown in Figure 3B. The number of GFAP-positive astroglial cells was significantly increased (versus control) in the contralateral SC at both 7 and 30 days and in the ipsilateral SC at 7 days after NMDA injection (Figure 3B). No morphological differences in the SC were observed among the control and sham-treated mice (data not shown).

### BDNF Expression in SC Cells

**Figure 4:**
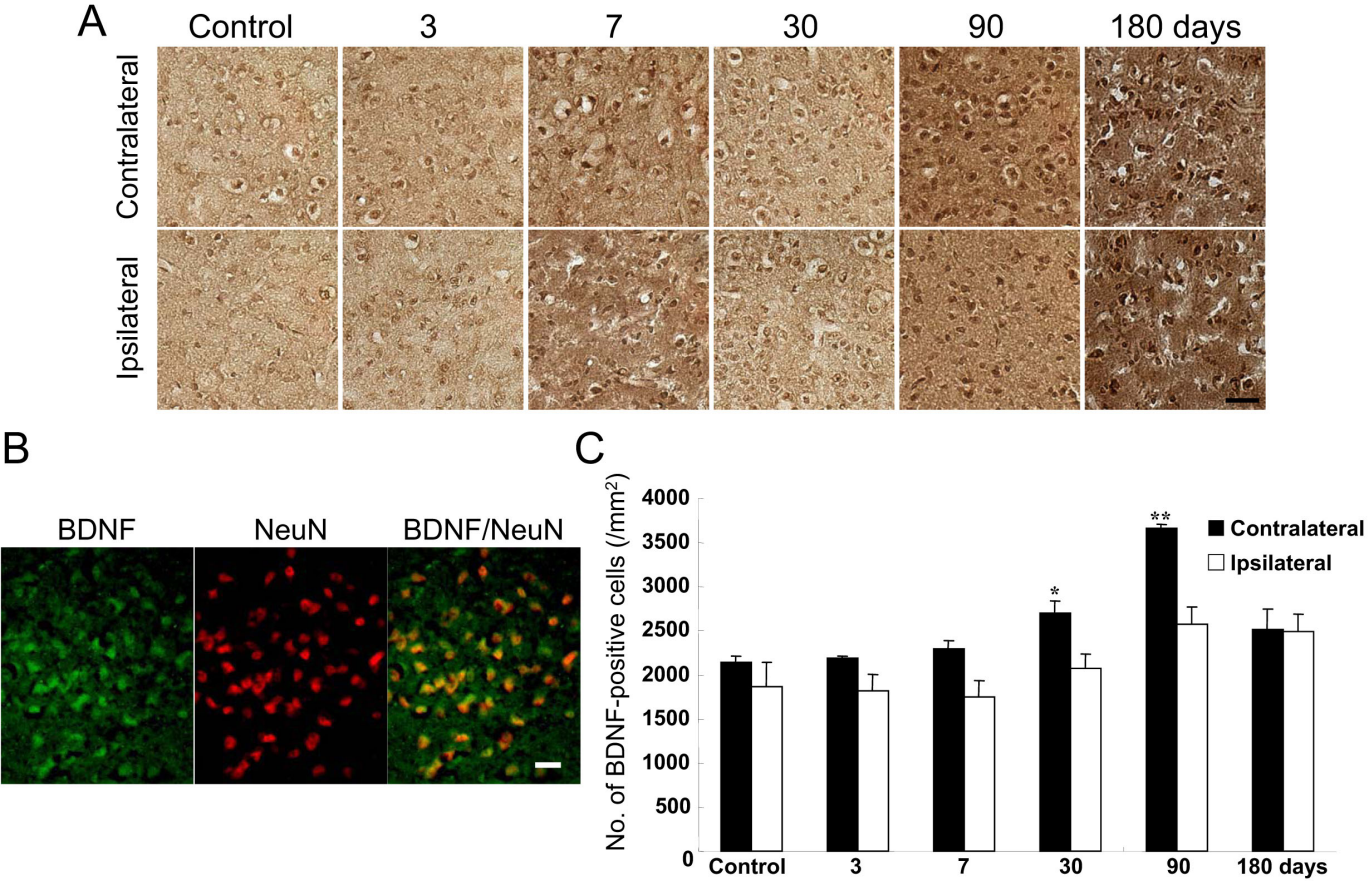
BDNF-positive cells of sections of the SC. **A:** Representative microphotographs of the superior colliculus (SC; the contralateral side and the ipsilateral side) are shown for the control group (untreated) 3, 7, 30, 90, and 180 days after *N*-methyl-D-aspartate (NMDA) injection. The scale bar represents 30 µm. **B:** Representative photographs show brain-derived neurotrophic factor (BDNF) and neuronal nuclear specific protein (NeuN) immunostaining, and BDNF/NeuN double-immunostaining of the contralateral SC at 30 days after intravitreal NMDA injection in mice. Some BDNF-expressing cells were colocalized with NeuN-positive neuronal cells, as indicated by the yellow color. The scale bar represents 20 µm. **C:** The average number of BDNF immunopositive puncta was counted in the SC. Each value represents the mean±SEM (n=3). The asterisk indicates p<0.05, while the double asterisk represents p<0.01 versus control (untreated mice; Dunnett’s test).

The specificity of the antibody against BDNF employed in this experiment has been characterized using immunohistochemistry and western blotting [24]. The antibody was unique to BDNF and did not detect other neurotrophins. To examine the time-dependent changes in BDNF expression, we performed immunohistostaining (Figure 4). In the control mice, BDNF-positive cells were observed in SC (Figure 4A), and they were significantly increased (versus control) in the contralateral SC at 30 and 90 days after NMDA injection (Figure 4C). Similarly, a slight increase (versus control) was observed in the ipsilateral SC at 90 and 180 days (Figure 4C). To identify BDNF-positive cells, we performed double immunofluorescence for BDNF and NeuN using the SC sections. Some NeuN-positive neuronal cells expressed BDNF at 30 days after NMDA injection (Figure 4B). No morphological differences in the SC were observed among the control and sham-treated mice (data not shown).

### Double immunofluorescence

**Figure 5:**
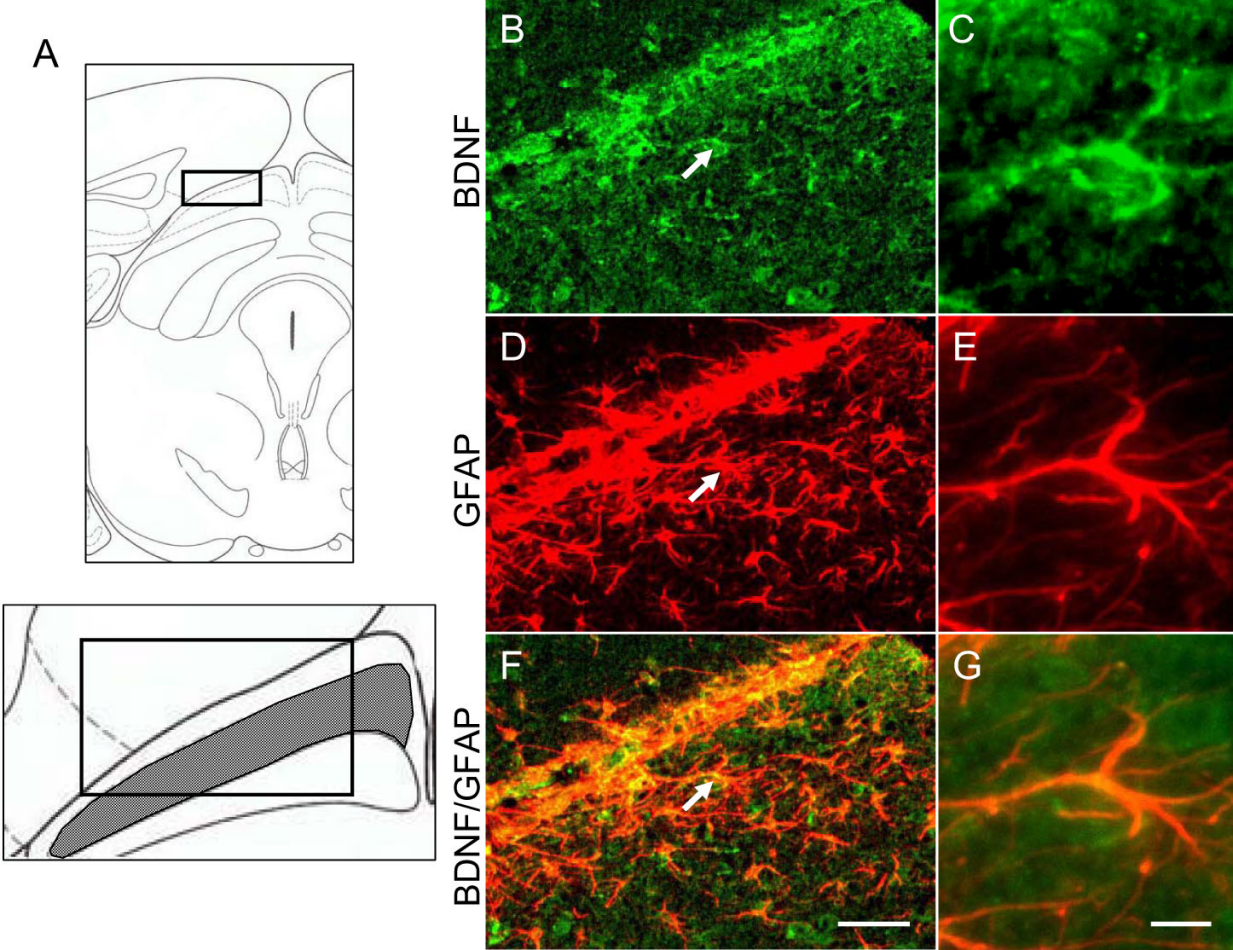
Colocalization of BDNF with GFAP by double immunofluorescence. **A:** Schematic drawing shows the coronal section through the level of the superior colliculus (SC; bregma −3.40 mm) in mice. The boxed area is the region of the superficial layer in the contralateral SC. Representative photographs show brain-derived neurotrophic factor (BDNF; **B** and **C**) and glial fibrillary acid protein (GFAP; **D** and **E**) immunostaining, and BDNF/GFAP (**F** and **G**) double-immunostaining of the contralateral SC at 30 days after intravitreal *N*-methyl-D-aspartate (NMDA) injection in mice. Some BDNF-expressing cells (**B** and **C**, green) were colocalized with GFAP-positive astroglial cells (**D** and **E**, red), as indicated by the yellow color in **F** (merge of **B** and **D**) and **G** (merge of **C** and **E**). The scale bars represents 50 µm (**B, D**, and **F**) or 10 µm (**C, E**, and **G**).

To identify other cells that were positive for BDNF, we performed double immunofluorescence for BDNF and GFAP in brain sections containing the SC after the intravitreal NMDA injection. Some GFAP-positive astroglial cells exhibited BDNF in the superficial layer of the contralateral SC at 30 days after NMDA injection (Figure 5).

## Discussion

In our previous study (also on mice), we observed a time-dependent decrease in the number of RGC after NMDA injection, with the damage first being evident at one day and almost reaching a plateau at three days after the injection [10]. These findings suggest that the neuronal degeneration we detected here in the contralateral SC was secondary to NMDA-induced retinal damage. It has also been reported that removal of the SC (in neonatal Wistar rats) results in a rapid loss of RGC, an event involving NMDA receptors [27,28]. It is likely that, because of interactions between RGC and the SC, the aforenamed types of neuronal degeneration result from a reduction in mutual stimuli and from a lack of neurotrophins (due to a dysfunction of anterograde transport from RGC). In mice, most of the optic fibers are crossed (70%–80%), and the direct retinohypothalamic projection to the contralateral SC is three times greater than that to the ipsilateral SC [29].

Astroglial activation is mainly characterized by marked overexpression of the major component of the gliofilaments, GFAP; this is a well known characteristic marker of astrocyte activation/reactive astrocytosis. Previous studies have revealed that transection of the optic nerve (or enucleation) leads to both fiber and terminal degeneration (Wallerian degeneration) within the SC [30-32] and to reactive changes in astrocytes [33,34]. Moreover, it has been reported that in rats, increases in GFAP within the SC follow 3, 3′-iminodipropionitrile-induced retinal degeneration, which is known to disrupt neurofilaments and axonal transport [35]. In the present study, GFAP expression was increased in the contralateral SC at 7, 30, and 90 days after unilateral intravitreal injection of NMDA. In addition, significant increases in GFAP expression were observed in the ipsilateral SC at 7 days. These findings indicate that the neuronal degeneration within the SC occurred not only contralaterally but also ipsilaterally, because of the nature of the projection from the retina (see Figure 1). Since damage to neuronal cells within the SC was not detected during the period of increased GFAP expression, reactive astrocytes may provide crucial support for neurons during this period through such functions as the production of trophic factors and the elimination of toxins [36,37].

The expression of neurotrophins is regulated by neuroelectric activity, and neurotrophins may modulate the efficacy of synaptic transmission, the growth of dendrites and axons, or the production of the structural elements necessary for synaptogenesis [38-42]. BDNF, a member of the neurotrophin family, plays a critical role in the development, survival, and synaptic plasticity of neurons within the adult central nervous system [38,43]. BDNF is synthesized by neurons and then anterogradely transported. Reactive astrocytes have been shown to produce BDNF [44-47]. In the present study, we observed significant increases in BDNF-positive cells in the contralateral SC at 30 and 90 days after NMDA injection, and at least a partial overlap with the cells showing increased expression of GFAP. These findings, indicating that BDNF was produced by GFAP-positive astroglial cells, are consistent with BDNF from reactive astroglial cells. However, as mentioned, BDNF is known to be synthesized by neurons and then anterogradely transported [48-50], and to be transferred from neuron to neuron [51]. The synthesis of BDNF is increased in RGCs immediately after intravitreal injection of NMDA in rats [51]. BDNF injected into the retina may be internalized by retinal ganglion neurons and transported to the SC, where BDNF may be released and transferred to the postsynaptic neurons [52]. In the present study, therefore, the BDNF expression we detected may be derived from GFAP-positive astroglial cells and/or from retinal ganglion neurons.

In this experiment, we could not clarify the detailed role of BDNF expressed in the SC after NMDA injection. In a previous study, polysialylated neural cell adhesion molecule, which contributes to nervous system plasticity, was expressed in the normal adult murine SC after RGC injury and involved in attempted visual system remodeling [53]. The BDNF gene transferred into RGC by electroporation or BDNF injected into the SC could prevent RGC loss after axotomy [54,55]. Further studies are needed to clarify the precise roles performed by BDNF in the SC during the processes leading to SC damage following NMDA injection.

In conclusion, we found that time-dependent morphological alterations and increases in the expression of BDNF occurred in the murine SC following NMDA-induced retinal damage. These findings may provide useful information concerning the pathological mechanisms of several disorders accompanied by retinal degeneration.
